# Medullary Thyroid Carcinoma: A Unique Case Report

**DOI:** 10.7759/cureus.64969

**Published:** 2024-07-20

**Authors:** Sumithra A, Lakshmi Priya Asokan, Vallal Kani, Volga Harikrishnan

**Affiliations:** 1 Department of Pathology, Saveetha Medical College and Hospitals, Saveetha Institute of Medical Sciences, Saveetha University, Chennai, IND

**Keywords:** fine needle aspiration cytology, endocrine, medullary thyroid cancer, calcitonin, thyroid carcinoma

## Abstract

Medullary thyroid carcinoma is a rare neuroendocrine tumor derived from parafollicular C-cells. It can be inherited as part of syndromes, such as familial medullary thyroid cancer (FMTC) and multiple endocrine neoplasia type 2 (MEN 2), or it can arise sporadically. We herein report a unique case of medullary thyroid carcinoma in a 50-year-old male who presented with a neck mass. Fine needle aspiration cytology (FNAC) of the thyroid and histopathological examination revealed a diagnosis of medullary thyroid carcinoma. Both carcinoembryonic antigen (CEA) and calcitonin are the key serum markers utilized in the diagnosis and monitoring of medullary thyroid cancer (MTC). Thorough evaluation, prompt identification, and efficient treatment constitute the pivotal measures for ensuring favorable survival outcomes.

## Introduction

Medullary thyroid carcinoma (MTC) is a type of cancer that originates from the parafollicular cells, commonly known as C cells, of the thyroid gland. These C cells secrete chromogranin and carcinoembryonic antigen (CEA), as well as the hormone calcitonin, which aids in controlling the body's calcium levels [1]. Medullary thyroid carcinoma (MTC) presents at a rate of approximately 5% of all thyroid tumors. It manifests either sporadically (in 75% of cases) or in a hereditary manner: Multiple endocrine neoplasia type 2 (MEN 2), familial MTC (FMTC) [2,3]. There are two forms of MEN 2 syndrome: Type 2A and 2B-germline mutations on chromosome 10q11 cause familial variants inherited by autosomal dominance. In addition to medullary thyroid carcinoma, pheochromocytoma, and parathyroid adenomas are more common in people with MEN 2A. However, medullary thyroid carcinoma, pheochromocytomas, marfanoid body habitus, and mucosal neuromas are associated with MEN 2B [4]. Because of its early dissemination and the lack of efficient systemic therapy, MTC has a bad prognosis compared to follicular and papillary thyroid cancers. Appropriate thyroid surgery and cervical nodal disease excision are the mainstay of MTC treatment. Following surgery, radiotherapy may improve local control [1]. Here, we report a case of medullary thyroid carcinoma diagnosed by fine needle aspiration cytology (FNAC) and a histopathological examination of total thyroidectomy with right radical neck dissection and a central compartment dissection specimen received at our institution. 

## Case presentation

**Figure 1:**
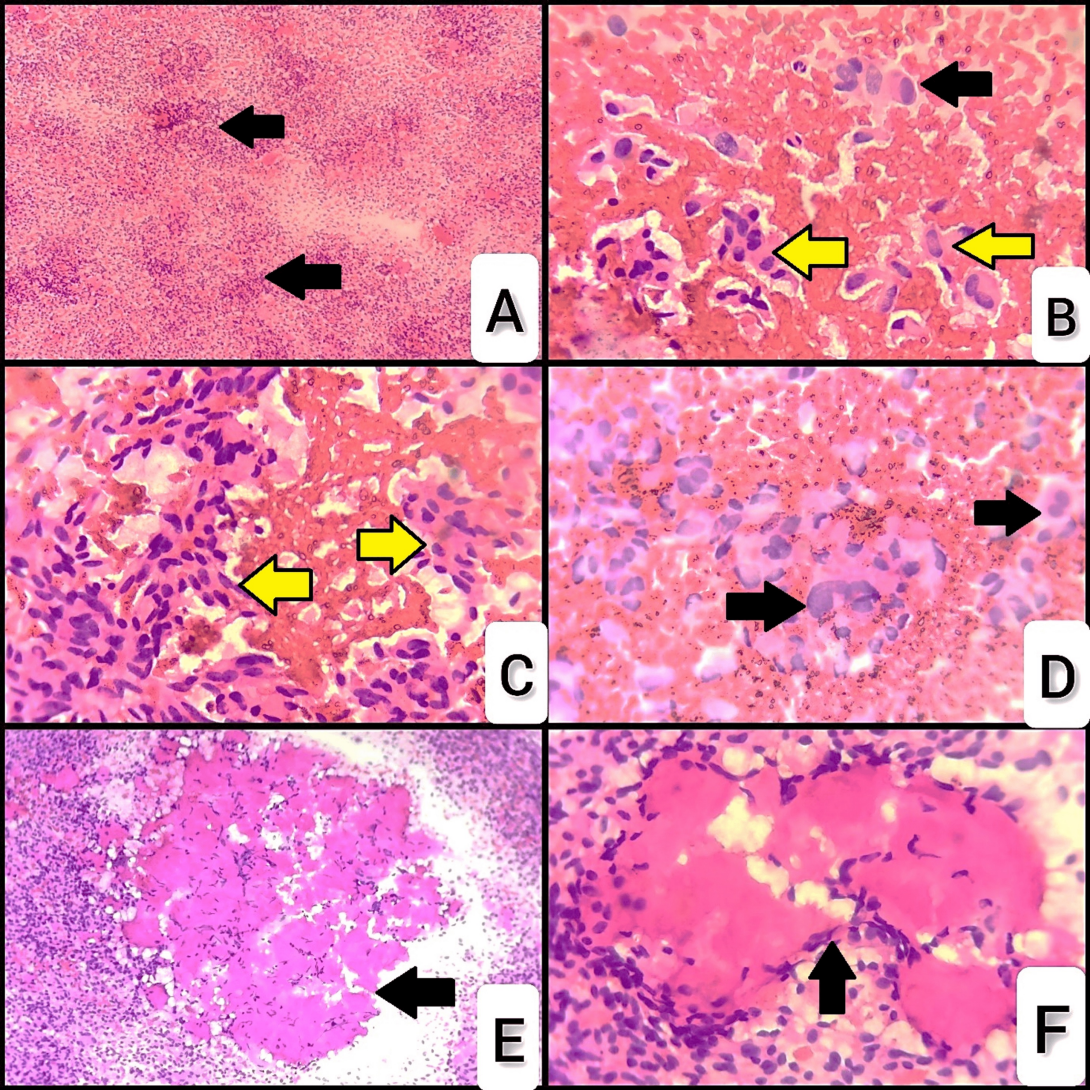
Microscopic features of medullary thyroid carcinoma of the right lobe of the thyroid in FNAC smears. (A) Cellular smears showing predominantly loosely cohesive clusters under 4X (arrows), (B) and (C) spindle cells with moderate pleomorphism (yellow arrows) admixed with occasional plasmacytoid cells (black arrow) under 40X, (D) few bizarre nuclei and binucleate cells in a background of RBCs (arrows)-40X, (E) and (F) amorphous eosinophilic stromal material (amyloid) amidst the spindle cells (arrows). FNAC: fine needle aspiration cytology.

A 50-year-old man complained of swelling on the right side of his neck over the last two years following an accidental injury, which gradually progressed to the size of 3 x 2 cm. There were no complaints of hoarseness of voice/sudden change in voice/stridor/dysphagia/heat intolerance/palpitations/tremors/protrusion of eyes/loss of weight/loss of appetite. There was no relevant family history. On inspection, a 3 x 2 cm swelling that moves with deglutition was found in the right lobe of the thyroid gland. Adjacent to this is a multilobulated 8 x 4 cm swelling, situated immediately lateral to the right-sided swelling, which doesn't move with deglutition. Dilated veins were seen surrounding the swelling on the right side. There were no signs of redness, previous scars, or sinus tracts. On palpation, it was firm in consistency and moved to deglutition. Additionally, multiple indurated nodes, each measuring 2 x 3 cm, were detected in the right cervical region, involving levels 2a, 2b, 3, and 4. These nodes were also firm, mobile, and matted together, extending 2 cm below the right earlobe to the supraclavicular region. Trail’s sign revealed the prominence of the clavicular head of the sternocleidomastoid muscle. The serum level of carcinoembryonic antigen level was elevated, measuring 265 ng/ml. The thyroid function test showed normal results. The patient's other baseline investigations were normal, and the patient was in a euthyroid state. USG-guided fine needle aspiration cytology was done from the right lobe of the thyroid and right cervical lymph nodes. Smears from the right lobe of the thyroid are cellular with predominantly dyscohesive to loosely cohesive clusters (Figure 1A) of spindle cells with moderate pleomorphism and admixed with occasional plasmacytoid cells (Figures 1B, 1C)and a few giant cells with bizarre nuclei (Figure 1D) in a background of RBCs and amyloid (Figures 1E, 1F). The Bethesda System for Reporting Thyroid Cytopathology 2023 [5] reported it as positive for malignancy and suggestive of medullary thyroid carcinoma, category VI (malignancy).

**Figure 2:**
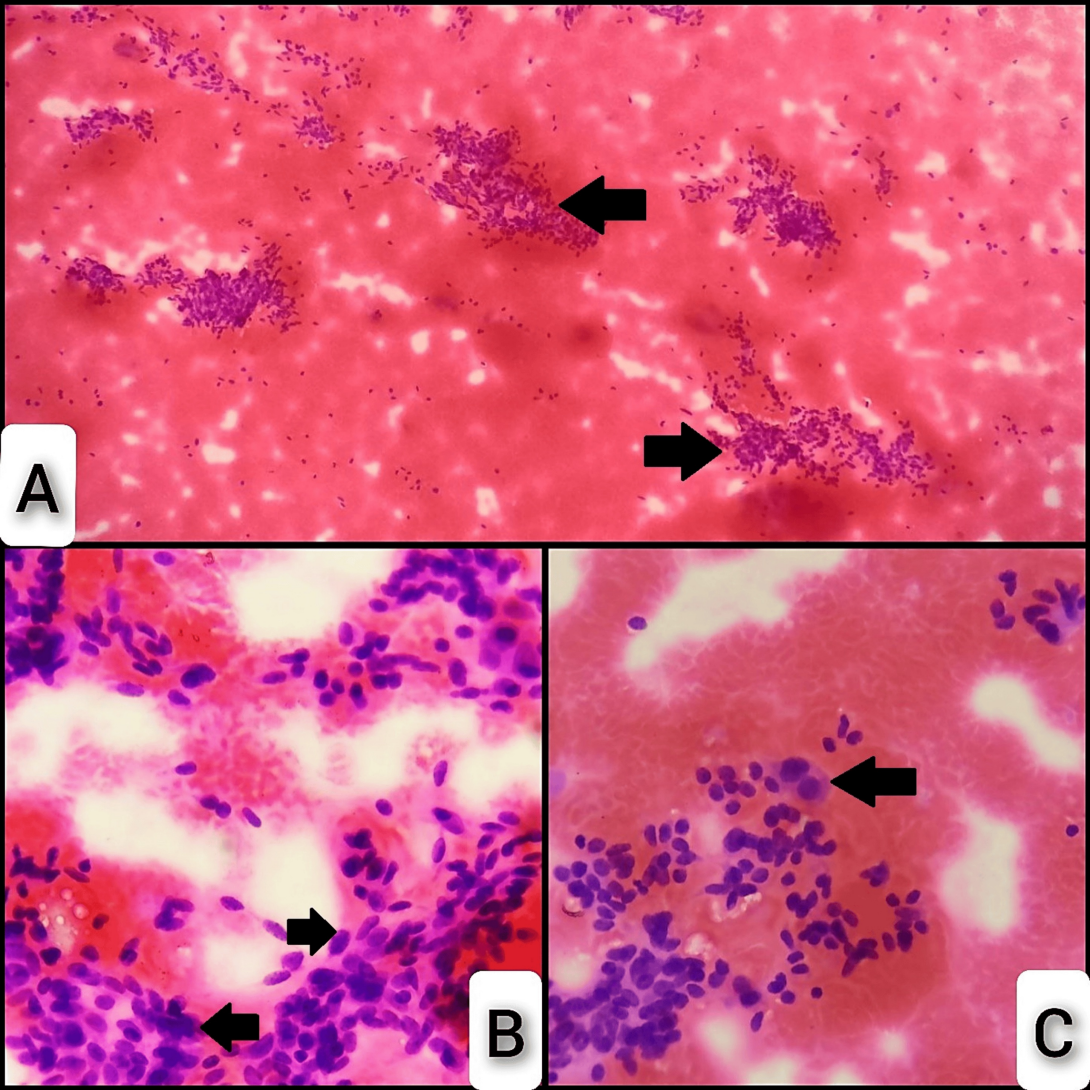
Microscopic features of metastatic carcinomatous deposits from the right cervical lymph node in FNAC smears. (A) Smears from the right cervical lymph node show loosely cohesive clusters under 4x (arrows), (B) and (C) spindle to oval cells along with a few binucleate cells showing pleomorphic nuclei in a hemorrhagic background under 40x (arrows). FNAC: fine needle aspiration cytology.

Smears from the right cervical lymph node show loosely cohesive clusters (Figure 2A) of the spindle to oval cells, with a few binucleate cells showing bizarre nuclei in a hemorrhagic background (Figures 2B, 2C), which was reported as positive for malignancy and metastatic carcinomatous deposits.

**Figure 3:**
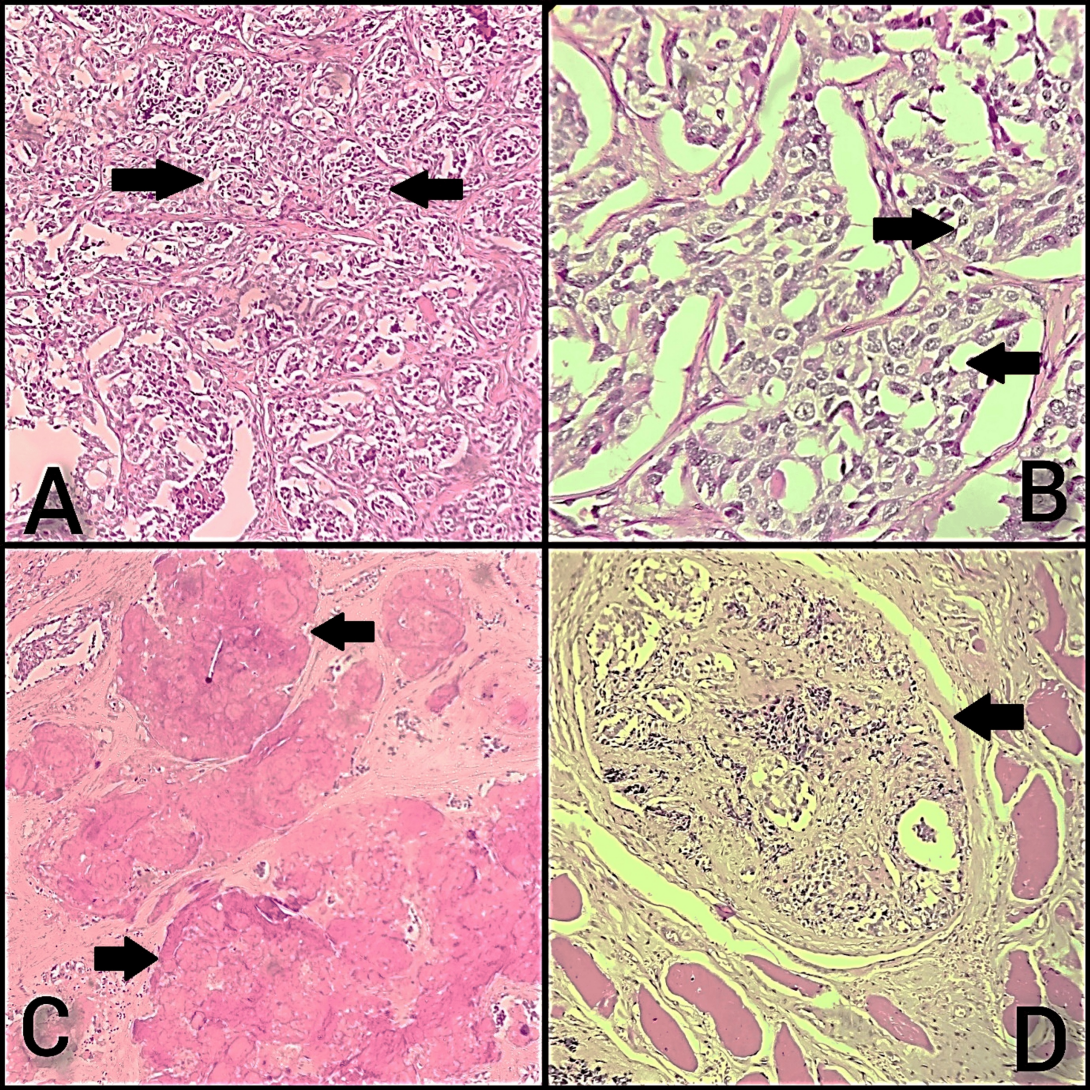
Microscopy of medullary carcinoma of thyroid. (A) An infiltrating malignant neoplasm arranged in nests and cords (arrows) (H&E)-4x, (B) nests of atypical oval to spindle cells with moderately pleomorphic hyperchromatic nuclei and moderate eosinophilic granular cytoplasm (arrows) (H&E)-40x, (C) stromal amorphous eosinophilic material deposition (amyloid) within the neoplasm (arrows), (D) extrathyroidal invasion of skeletal muscle (arrow).

**Figure 4:**
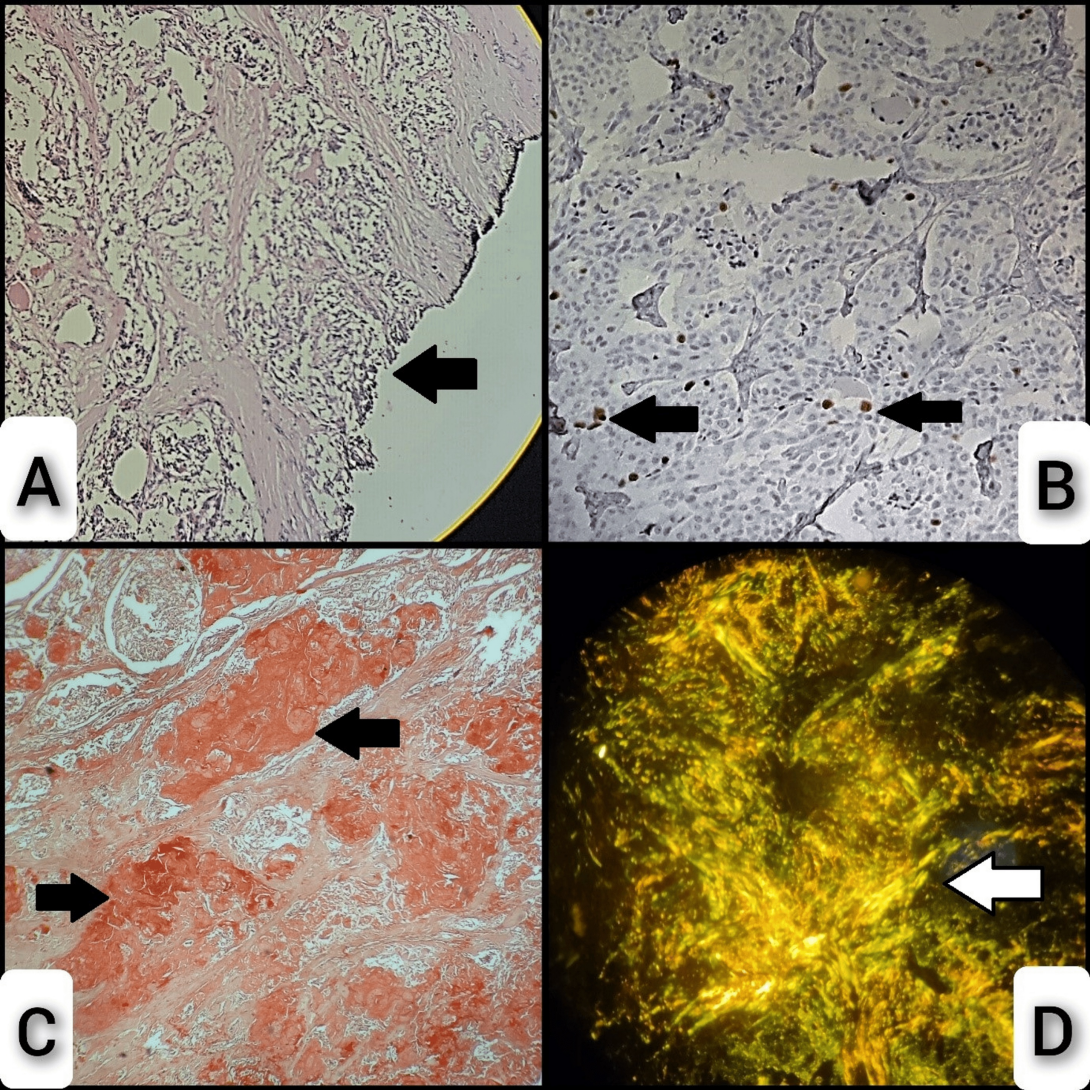
Light and polarized microscopy of medullary carcinoma of thyroid. (A) Resected margins showing positive for malignancy (arrow), (B) four percent of the tumor cells showing positivity for the cell proliferation marker Ki67 (arrows), (C) brick red positivity with Congo red stain depicting amyloid deposition (arrows), (D) apple-green birefringence under polarized microscope (arrow).

Subsequently, a total thyroidectomy with right radical neck dissection and central compartment dissection was done. Macroscopically, the right and left lobes of the total thyroidectomy specimen measured 5 x 3.5 x 2 cm and 4 x 2 x 1 cm, respectively, and the isthmus measured 2 x 1 cm. The external surface of the right lobe appeared nodular, while its cut surface revealed a gray-white to gray-tan lesion measuring 3.6 x 3 cm. The external surface of the left lobe appears bosselated, and the cut surface shows gray-brown areas. The capsule appears to be breached grossly, and macroscopic extrathyroidal extension was noted. Microscopically, multiple sections showed thyroid parenchyma with an infiltrating malignant neoplasm arranged in nests and cords (Figure 3A), composed of oval to spindle-shaped cells with moderate eosinophilic granular cytoplasm and moderately pleomorphic hyperchromatic nuclei (Figure 3B). Also, focal areas show stromal amorphous eosinophilic material deposition within the neoplasm (Figure 3C). Extrathyroidal invasion of skeletal muscle was also noted (Figure 3D). Resected margins were positive for malignancy (Figure 4A) and seven out of fifteen lymph nodes were involved by malignancy. A Congo red special stain was utilized to identify amyloid, revealing a positive result with characteristic apple-green birefringence when viewed under a polarized microscope (Figures 4C, 4D). Cell proliferation marker Ki-67 antigen was done and showed positivity in four percent of the tumor cells (Figure 4B). According to the College of American Pathologists, AJCC 8th edition, our case was classified as pT3b (tumor of any size with gross extrathyroidal extension invading only strap muscles) and pN1b (metastasis to unilateral (right) lateral neck lymph nodes). Thus, the case was reported as medullary thyroid carcinoma, right side, pT3bN1b. Postoperatively, the serum level of calcium was 7.8 mg/dl, and the patient was discharged after being prescribed a 75-milligram dose of Thyronorm.

## Discussion

Medullary carcinoma of the thyroid is a rare subset of thyroid tumors, making up about 5% of total thyroid cancer cases. It is a malignancy of the parafollicular thyroid C cells, which secrete calcitonin, and often appears in the fourth or sixth decade of life. Gene mutations in the rearranged during transfection (RET) proto-oncogene may cause some hereditary MTC instances, even though more than 80% of MTC cases are sporadic [6]. The clinical manifestations of individuals with MTC can vary significantly. Typically, a palpable enlargement of the thyroid gland is detected on examination. It is commonly found that cervical lymph nodes have been affected by both sporadic and inherited forms of MTC at this stage [4]. Serum calcitonin levels indicate the presence of a tumor, except in patients who do not produce calcitonin. Medullary thyroid carcinoma typically shows positivity for carcinoembryonic antigen (CEA). Serum CEA levels can be monitored in non-calcitonin-secreting tumors to evaluate the extent of the disease and the efficacy of treatment [7]. Unless there is vascular or skeletal muscle invasion, radical neck dissection is not usually advised due to its significant morbidity and lack of effect on long-term survival [1]. It is typical to observe a mixture of spindled and epithelioid cells and cells with plasmacytoid morphology. The cytoplasm is finely granular because of neurosecretory granules present in varying amounts. The chromatin in neuroendocrine tumors is typically described as "salt and pepper" chromatin, consisting of a mixture of tiny and coarse particles.

Mitotic figures are often sparse, and cells can be organized in sheets, nests, trabeculae, or even follicles [7]. Sarcoidosis, benign masses such as lipomas or hemangiomas, or cervical lymphadenitis from infections could be the differential diagnosis for spontaneous MTC [4,8]. The inheritance pattern for hereditary MTC is autosomal dominant. As a result, it is highly recommended that patients with positive results are encouraged to test specific family members [6]. While some patients who present with advanced metastatic cancer have a long event-free disease period, others pass quite quickly from disease progression [9]. Based on the American Joint Committee on Cancer (AJCC)/Union for International Cancer Control (UICC) staging, Kloos et al. (2018) report a five-year survival of nearly 100% in stage I, 93% in stage II, 71% in stage III, and 21% in stage IV [10]. The extent and nature of the disease determine the course of treatment. Patients with MTC who receive early treatment and show no lymph node metastasis indicate a good prognosis and a low likelihood of recurrence. However, those with nodal disease are more prone to disease recurrence and progression [4]. According to the Society of Surgical Oncology, individuals with palpable lateral neck lymph nodes or involvement of the central neck compartment should undergo ipsilateral neck dissection for sporadic MTC. Following surgery, patients should follow up with proper surveillance of their calcitonin or CEA levels every three months. This can be done once a year [4]. Radioactive iodine therapy is not utilized in treating medullary thyroid carcinoma. This may be elucidated by the fact that, in contrast to other types of thyroid cancer, the parafollicular cells, the source of MTC, do not take up iodine. As a result, iodine scanning cannot be employed to assess the distant metastasis of MTC to other organs [4].

## Conclusions

Following a thyroidectomy, levothyroxine is indeed the most commonly prescribed medication, an analog of thyroid hormone, to keep the serum TSH levels within the range of euthyroid. To keep an eye out for any signs of a recurrence of disease or suspicion, routine PET/CT scans should be performed. Regularly checking the level of calcitonin in the serum assists in identifying medullary thyroid cancer (MTC) and monitoring its potential recurrence. The absence of elevated CEA and calcitonin levels does not eliminate the possibility of medullary thyroid carcinoma, warranting consideration for calcitonin-negative medullary carcinoma. FNAC is regarded as a first-line diagnostic test for the assessment of thyroid lesions. A significant increase in the cure rate of malignant neoplasms would result from early detection and therapy. In addition, histopathology and immunohistochemistry play crucial roles as tumor markers, essential for reaching an accurate diagnosis. 
